# Dapagliflozin inhibits ferroptosis to improve chronic heart failure by regulating Nrf2/HO-1/GPX4 signaling pathway

**DOI:** 10.1371/journal.pone.0317295

**Published:** 2025-01-28

**Authors:** Jing Zhang, Xuefeng Chen, Shilin Lv, Qingqing Hao

**Affiliations:** 1 Hebei North University, Zhangjiakou City, Hebei Province, P.R. China; 2 Hebei General Hospital, Shijiazhuang City, Hebei Province, P.R. China; 3 Hebei Medical University, Shijiazhuang City, Hebei Province, P.R. China; Second Xiangya Hospital, CHINA

## Abstract

**Objective:**

To study the effect of Dapagliflozin on ferroptosis in rabbits with chronic heart failure and to reveal its possible mechanism.

**Methods:**

Nine healthy adult male New Zealand white rabbits were randomly divided into Sham group (only thorax opening was performed in Sham group, no ascending aorta circumferential ligation was performed), Heart failure group (HF group, ascending aorta circumferential ligation was performed in HF group to establish the animal model of heart failure), and Dapagliflozin group (DAPA group, after the rabbit chronic heart failure model was successfully made in DAPA group). Dapagliflozin was given by force-feeding method. Echocardiography was used to assess cardiac function, HE staining to evaluate pathological changes in the heart, Prussia blue staining to observe iron ions in myocardial tissue, and enzyme-linked immunosorbent assay (ELISA) to determine serum levels of the inflammatory factors interleukin-1β (IL-1β), interleukin-6 (IL-6), and tumor necrosis factor-α (TNF-α) at the end of week 12 and/or the end of week 16. The oxidative stress related indexes of malondialdehyde (MDA), superoxide dismutase (SOD) and superoxide dismutase (GSH-Px) in serum were quantitatively analyzed by colorimetry. Protein expression levels of nuclear factor E2-related factor 2(Nrf2), heme oxygenase-1(HO-1), glutathione peroxidase 4(Gpx4) were detected by Western blot.

**Results:**

In animals with chronic heart failure, Dapagliflozin improved cardiomyocyte hypertrophy, degeneration and necrosis. Dapagliflozin increased serum GSH-Px and SOD levels and decreased IL-1β, IL-6, TNF-α and MDA levels (P < 0.05) in a rabbit model of heart failure. Dapagliflozin also decreased cardiac iron ion levels and increased Nrf2, HO-1 and GPX4 protein expression.

**Conclusion:**

Dapagliflozin can improve heart failure by inhibiting oxidative stress and ferroptosis, and its mechanism may be related to the regulation of Nrf2/HO-1/GPX4 signaling pathway.

## 1 Introduction

Heart failure (HF) is a group of clinical syndromes that are the end stage of cardiac disease progression, with high prevalence, mortality, and re-hospitalization rates. Chronic heart failure (CHF) is an advanced stage of various heart diseases, and it is the most important cause of death in many cardiovascular diseases progressing to the end stage, which has caused a serious medical burden worldwide [1, 2]. According to a survey, it is known that the incidence of HF in China has increased by 44% in the last 15 years, and the incidence of HF in people over 35 years old is 1.3% [3]. Clinical treatment of CHF is mainly treated with a combination of diuretics, cardiotonic agents, vasodilator drugs, and β-blockers. Although the standardized drug treatment of heart failure is becoming more and more effective, its mortality and rehospitalization rates remain high [4]. Heart failure is now a serious threat to human life and health, and has become one of the most challenging problems of cardiovascular disease in the 21st century. It has become a challenging problem that doctors are constantly researching on how to effectively treat HF and reduce the mortality rate.

Sodium-glucose co-transporter 2(SGLT-2) is mainly expressed in the kidney and small intestine, and works with the glucose transporter to control the reabsorption of glucose by the kidney through the action of sodium-potassium pump [5]. Sodium-glucose co-transporter 2 inhibitors(SGLT2-i) are a new type of hypoglycemic drug. By competitively inhibiting SGLT-2 in S1 segment of proximal convoluted tubules, it can effectively block the reabsorption of glucose and sodium ions by proximal convoluted tubules, and promote the effective excretion of glucose by renal tubules, thus effectively regulating blood glucose levels [6]. In recent years, researchers have found that SGLT2-i originally used for the treatment of diabetes have potential effects in the treatment of heart failure, among which dapagliflozin not only meets the clinical hypoglycemic needs, but also has an obvious role in cardiovascular aspects. The study found that the addition of dapagliflozin to standard treatment for heart failure reduced the risk of cardiovascular death in patients with heart failure regardless of comorbidities [7]. As clinical trial results continue to be published, dapagliflozin has become a first-line therapy in the field of HF, but its exact molecular mechanism remains unclear [8].

Iron is an essential trace element for human body [9], playing a critical role in body homeostasis. In the body, iron typically exists in either the ferrous (Fe^2^⁺) or ferric (Fe^3^⁺) forms, and is absorbed in the upper end of the duodenum and jejunum during the circulation process. Under the action of ceruloplasmin, ferrous iron in the blood is oxidized to ferric iron, which is then bound with ferritin and transported to various tissues. Ferroptosis, first proposed by Dixon in 2012 [10], is a novel form of regulated cell death that is dependent on iron ions. During the occurrence of ferroptosis, overloaded iron accumulates in cells to generate reactive oxygen species (ROS), which oxidize polyunsaturated fatty acids and cause disruption of cell membrane structure, ultimately leading to cell death. In recent years, the role of ferroptosis in the occurrence and development of cardiovascular diseases has attracted more and more attention, and understanding the relationship between ferroptosis and heart-related conditions is of great importance to explore new intervention targets and strategies for cardiovascular ailments.

Previous studies by our research group have shown that dapagliflozin has a good therapeutic effect on rabbits with chronic heart failure, but the mechanism of inhibiting ferroptosis and improving chronic heart failure through the regulation of the Nrf2/HO-1/GPX4 signaling pathway remains unclear. The aim of this study is to investigate the regulatory effects of Dapagliflozin on pressure overload-induced heart failure in rabbits and the Nrf2/HO-1/GPX4 signaling pathway from the perspectives of oxidative stress and ferroptosis, in order to explore the potential mechanisms and intervention strategies of dapagliflozin.

## 2 Materials and methods

### 2.1 Animals

Nine healthy New Zealand white rabbits (6 months old, weight: 2.5–3.5 kg, both male and female rabbits) were provided by the Experimental Animal Center of Hebei Medical University. After the animals were purchased, they were fed in separate cages at the Clinical Medical Research Center of Hebei Provincial People’s Hospital, with room temperature (22±2)°C, humidity 40%-70%, free to ingest food and water, avoiding noise. The rabbits were allowed to acclimate to the environment for one week before being used for the experiment.

### 2.2 Main reagents and instruments

The IL-1β, IL-6, and TNF-α detection kits were purchased from Wuhan USCN Business Co., Ltd. The MDA content detection kit and SOD activity detection kit were obtained from Beijing Biosea Biotechnology Co., Ltd. The GSH-Px colorimetric assay kit and ferrous ion colorimetric assay kit were purchased from Wuhan Elabscience Biotechnology Co., Ltd. The GPX4 polyclonal antibody (67763-1-Ig) and HO-1 polyclonal antibody (66743-1-Ig) were acquired from Wuhan Sanying Biotechnology Co., Ltd. The Nrf2 polyclonal antibody (bs-1074R) was obtained from Beijing Bioss Biotechnology Co., Ltd. The EPIQ7 ultrasound diagnostic system was purchased from Philips Corporation in the United States. The -80°C ultra-low temperature freezer was purchased from Haier Qingdao. The PVDF membrane was obtained from Millipore, USA. The high-speed centrifuge was sourced from Eppendorf, a company based in Germany. The electrophoresis apparatus, gel glass plates, clamps, vertical electrophoresis tanks, and transfer tanks were all from Beijing Liuyi Instrument Factory.

### 2.3 Methods

#### 2.3.1 Animal experimentation methods and ethical considerations

In this study, we followed strict ethical guidelines and experimental animal welfare standards to ensure the welfare of the animals during the experiment and the reliability of the experimental results. Anesthesia and analgesia are essential components of animal experimentation, as they ensure that animals do not experience pain or discomfort during procedures. We used intravenous agents to provide reliable sedation and analgesia, allowing for safe and effective procedures. The animals were closely monitored for signs of distress, and the dosage was adjusted as necessary to maintain a stable anesthetic state. At the end of the experiment, we adopted a humane and ethical method of sacrifice. We euthanized all the animals with an intravenous overdose of sodium pentobarbital, ensuring a painless death.

The animals’ behavior was closely observed throughout the study. Any signs of distress, such as abnormal vocalizations, changes in activity levels, or signs of aggression, were immediately addressed. Behavioral monitoring allowed us to intervene promptly and provide appropriate care to alleviate suffering.

#### 2.3.2 Measures to alleviate pain in animals

When constructing the rabbit model of chronic heart failure, it was very important to select the appropriate intravenous anesthetic and analgesic to relieve the pain of experimental animals and ensure the smooth conduct of the experiment. Experimental animals were routinely fasted 8 hours before surgery and weighed before surgery. The experimental animals were anesthetized with 1ml/kg injection of 3% pentobarbital through the auricular vein. After the pedal-off reflex of the hind limbs disappeared and the pain stimulation did not respond, the experimental animals were fixed in the supine position on the animal operating table for operation. During the operation, the state of anesthesia was checked at any time, and anesthetic was added in time if necessary.

#### 2.3.3 Ethics statement

This study has been approved by the Medical Ethics Committee of Hebei Provincial People’s Hospital (Ethical Review No. 2024-DW-075). All participants provided informed written consent.

#### 2.3.4 Experimental grouping and animal model establishment

After one week of adaptive feeding, 9 experimental animals were randomly divided into sham operation group (Sham group, N = 3), heart failure group (HF group, N = 3) and dapagliflozin group (DAPA group, N = 3). The rabbits in the sham group had their thoracic cavity closed immediately after thoracotomy without ascending aortic ring ligation, and were given 2 ml of saline orally by force-feeding every day for 10 weeks after 12 weeks of normal postoperative rearing. The animals in the HF group and the DAPA group were treated with ascending aortic annuloplasty (annular ligation of the ascending aorta 1 cm from the root of the ascending aorta, measurement of the circumference of the aorta by the length of the silk thread, so that the circumference of the ascending aorta was shortened to 60% of the original circumference) to increase the afterload of the heart, in order to establish a heart failure animal model. After the operation, the rabbits were raised normally and the general conditions of each group were evaluated at the end of the 12th week. The ultrasound examination of the heart of the HF group and the DAPA group fulfilled the diagnostic criterion of EF<40%, and at the same time, they showed the clinical manifestations of heart failure, such as decreased food and water intake, slow body weight gain, slow response, and shortness of breath, which suggested that the model of heart failure had been successfully established. Starting from the 13th week after surgery, the rabbits in the HF group were given saline 2ml per day orally by force-feeding for 10 weeks, and rabbits in the DAPA group were given dapagliflozin (1 mg/kg/day) by force-feeding orally in a volume of about 2ml per day for 10 weeks.

#### 2.3.5 Echocardiography test

In the 22nd week of the experiment, which is the 10th week of drug treatment, echocardiographic assessment was performed on the each group of experimental animals. The animals were positioned in the supine position with their chest shaved to expose the skin, and the left ventricular long-axis section was taken on the left side of the sternum, and the M-ultrasound mode was activated to determine the interventricular septum (IVS), the left ventricular end-diastolic diameter (LVEDD), the left ventricular end-systolic diameter (LVESD), the left ventricular posterior wall (LVPW), and the left ventricular ejection fraction (LVEF) via M-ultrasound curves. Simultaneously, an electrocardiogram was recorded to measure the heart rate of the experimental animals in each group. Measurements were collected for at least three consecutive cardiac cycles, and the average of these three measurements was used for statistical analysis.

#### 2.3.6 HE staining to observe cardiac histopathology

After the paraffin sections were dewaxed and rehydrated, the sections were sequentially placed in hematoxylin staining solution and eosin staining solution for staining. The results of myocardial tissue staining were observed under a light microscope.

#### 2.3.7 Iron test kit to measure serum iron levels in each group of rabbits

Took rabbit serum samples, mixed well with buffer and set aside. Added the samples sequentially according to the operating instructions of the iron kit, mixed well, incubated at 37°C for 10min, measured the OD value of each well at 593nm on the enzyme labeling instrument, and calculated the iron content of the rabbit serum samples.

#### 2.3.8 Measurement of serum inflammatory factor levels by ELISA

The levels of inflammatory factors IL-1β, IL-6 and TNF-α in rabbit serum were quantified by double-antibody sandwich ELISA in strict accordance with the instruction manual of the kit. In the predetermined positions of the microplate, the standard and serum samples to be tested were added, incubated at 37°C for 1 hour, the liquid was discarded, and the plate was shaken dry. The detection solution A working solution provided in the kit was added, and continued incubation at 37°C for 1 hour. After washing, the detection solution B working solution provided in the kit was added, incubated at 37°C for 30 minutes. After washing, 90μL of 3,3’,5,5’-Tetramethylbenzidine Substrate Solution (TMB) was added to each well, incubated at 37°C in the dark for color development. Finally, 50μL of stop solution was added to each well to terminate the reaction, and the absorbance value was read using an enzyme-labeled instrument. The concentration of the inflammatory factor was calculated through the standard curve.

#### 2.3.9 Colorimetric assay to detect oxidative stress-related indicators in serum

In the 22nd week of the experiment, which is the 10th week of drug treatment, after the cardiac ultrasound examination, the animals were anesthetized and fixed on a small animal experimental table. After thoracotomy, blood was quickly drawn from the abdominal aorta by needle puncture. The blood was then centrifuged using a high-speed centrifuge, labeled, and stored in a -80°C freezer for subsequent testing. Serum samples were taken from rabbits and the levels of ROS, MDA and GSH were determined using the appropriate test kits according to the instructions.

#### 2.3.10 Detection of protein expression levels of Nrf2, HO-1, and GPX4 by Western blot

The expression levels of proteins related to Nrf2/HO-1/GPX4 signaling pathway in cardiac tissue were measured using western blot protein blotting technique. The extracted total protein samples from cardiac tissue were removed from -80°C refrigerator and immediately inserted into ice (to reduce protein degradation) to be melted. Determined the protein concentration by Caulmers Brilliant Blue method, according to the result of protein quantification, added 20μg of the corresponding volume of the total protein sample with 5× protein gel electrophoresis uploading buffer, gently mixed, denatured at 95°C for 10 minutes, and immediately inserted into the ice to be used. The 20ug protein sample was gently added to the gel wells and the proteins were separated by SDS-PAGE gel electrophoresis. After gel electrophoresis, the protein bands separated on the gel were transferred to the PVDF membrane by transfer electrophoresis, and after transferring the membrane, the transferred membrane was put into the sealing solution and sealed at room temperature with slow shaking on a shaker for 1 h. The sealed membrane was put into the working solution of Nrf2, HO-1, and GPX4 primary antibody (diluted concentration of 1:1000) directly, and then washed after the reaction at 4°C for the night. The washed primary antibody was put into horseradish peroxidase-labeled goat anti-rabbit secondary antibody working solution (1:10,000) at room temperature and protected from light with slow shaking action for 60 minutes, and the washed membrane was exposed with Tennent T4600 chemiluminescence instrument, and the gray value was analyzed by image J software.

### 2.4 Statistical analysis

Data processing in this study was performed using SPSS23.0 software. All data were expressed as mean ± standard deviation $\bar{X}$±S, and if the data of multiple groups conformed to normal distribution and variance chi-square, one-way ANOVA was used, and further two-by-two comparison of differences between groups was performed by LSD-t test, and the difference was statistically significant at P<0.05. If the data of multiple groups conformed to normal distribution but the variance was not aligned, Welch’s test was used, and further two-by-two differences between groups were compared using Dunnet’s T3 test, and the difference was statistically significant at P<0.05. If the data did not conform to normal distribution, non-parametric test was used, and Kruskal-Wallis test was used, and the difference was statistically significant at P<0.05.

### 2.5 Technical route

**Fig 1:**
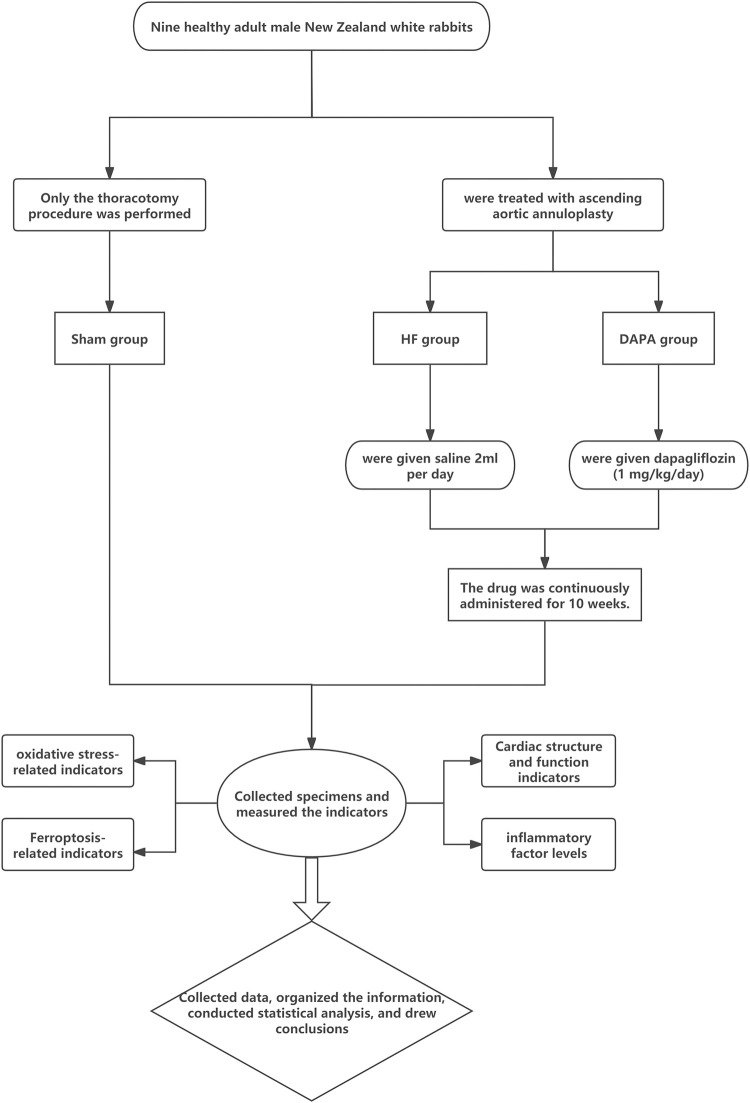
Flowchart of the entire experimental process.

We have meticulously crafted a comprehensive mind map to encapsulate the entire experimental process. This visual representation not only delineates each step with clarity but also enhances understanding through its structured and detailed layout. The mind map serves as a navigational tool, guiding us through the intricate journey from hypothesis formulation to data analysis, ensuring that every phase is executed with precision and efficiency (Fig 1).

## 3 Results

### 3.1 The changes of structural and cardiac function in the experimental rabbit heart

**Fig 2:**
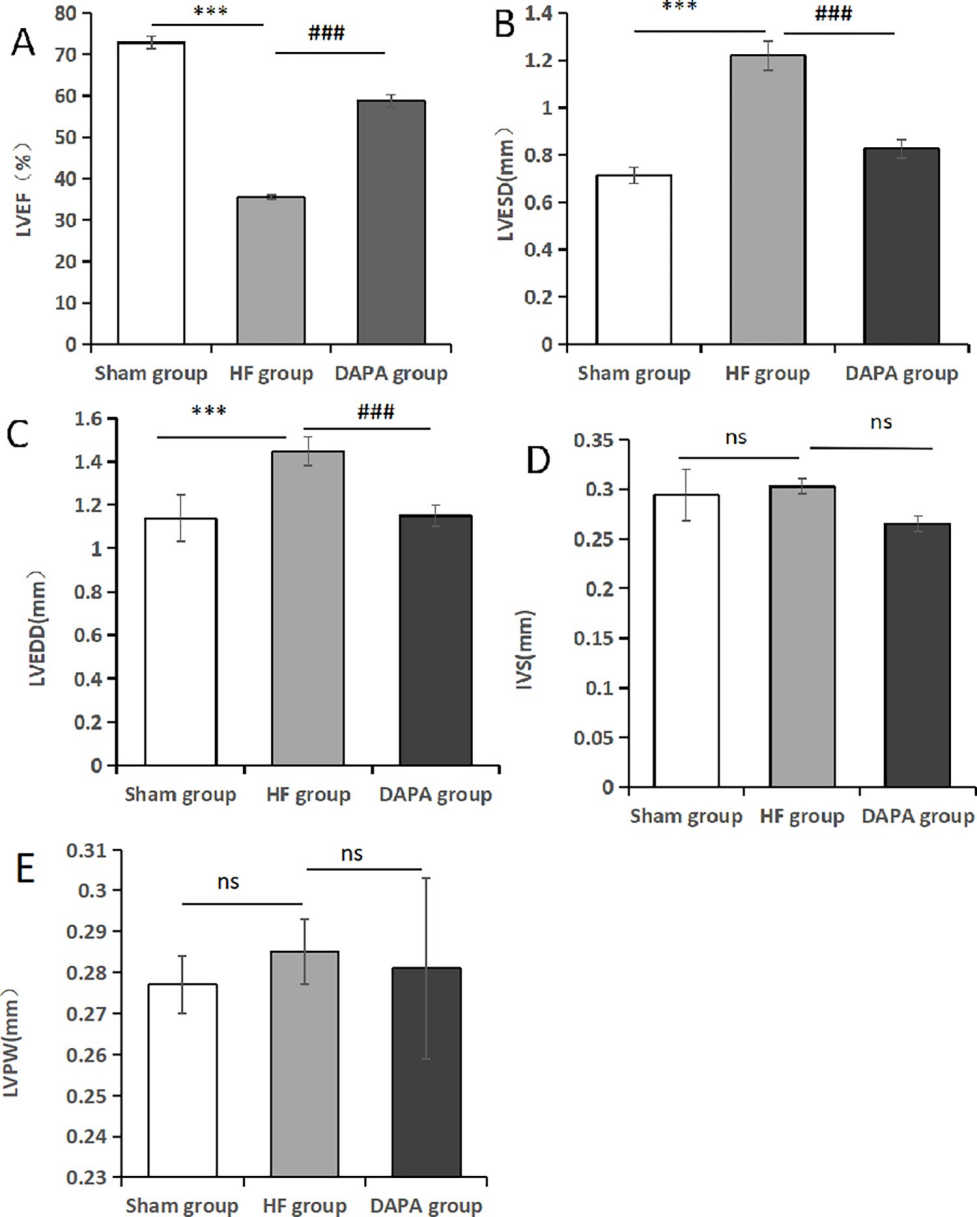
Echocardiography results of each group of rabbits. Note: (A). LVEF; (B). LVESD; (C). LVEDD; (D). IVS; (E). LVPW; *Compared with the Sham group, *P<0.05, **P<0.01, ***P<0.001; ^#^compared with the HF group, ^#^P<0.05, ^##^P<0.01, ^###^P<0.001.

After 10 weeks of drug treatment, there was no statistically significant difference between IVS and LVPW in experimental animals in the sham, HF, and DAPA groups (P > 0.05). Compared with the sham operation group, LVESD and LVEDD increased and LVEF decreased in the HF group, and the difference was statistically significant (P<0.05). Compared with the HF group, LVESD and LVEDD decreased and LVEF increased in the DAPA group, and the difference was statistically significant (P<0.05) (Table 1, Fig 2).

**Table 1:** Echocardiography results of each group of rabbits ($\bar{X}$±S).

	Sample size	LVEF	LVESD	LVEDD	IVS	LVPW
		(%)	(mm)	(mm)	(mm)	(mm)
**Sham group**	3	72.933±1.55	0.716±0.034	1.137±0.107	0.294±0.026	0.277±0.007
**HF group**	3	35.467±0.473*	1.22±0.06*	1.447±0.067*	0.303±0.008	0.285±0.008
**DAPA group**	3	58.8±1.609^#^	0.827±0.039^#^	1.15±0.05^#^	0.265±0.008	0.281±0.022

Note: Compared with the Sham group

*p<0.05; compared with the HF group

^#^p<0.05.

**Fig 3:**
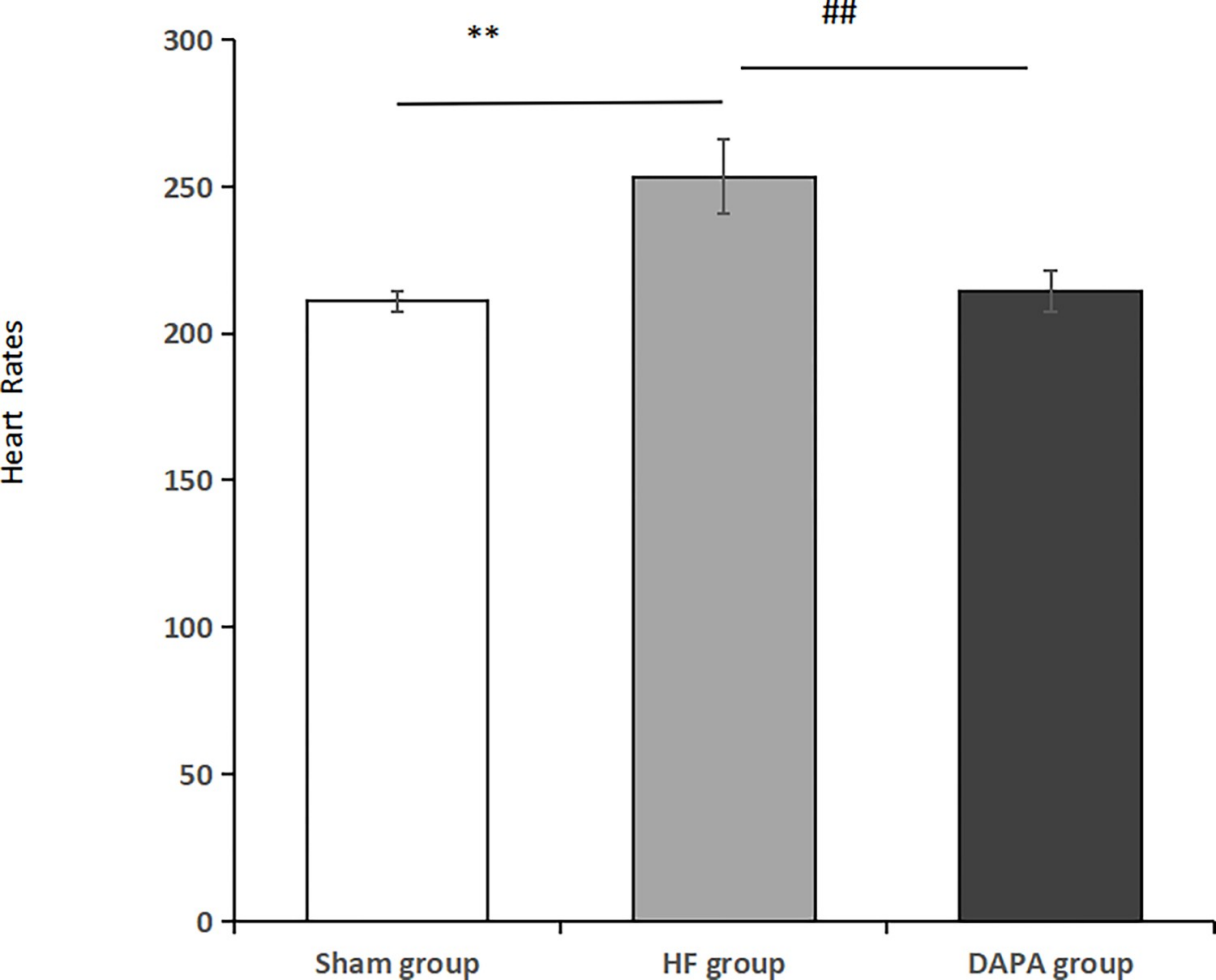
Heart rate changes in each group of rabbits. Note: **Compared with the Sham group, **P<0.01;##compared with the HF group, ##P<0.01.

Compared with the sham operation group, the heart rate of the HF group increased, and the difference was statistically significant (P<0.05), and after 10 weeks of drug intervention, the heart rate of the dagliflozin group decreased compared with the HF group, and the difference was statistically significant (P<0.05) (Table 2, Fig 3).

**Table 2:** Comparison of heart rates among each group of rabbits ($\bar{X}$±S).

Group	Sample size	Heart Rates
Sham group	3	211±3.606
HF group	3	253.333±12.503*
DAPA group	3	214.333±7.024^#^

Note: Compared with the Sham group

*p<0.05; compared with the HF group

#p<0.05.

### 3.2 Comparison of myocardial histopathological damage among each group of rabbits

**Fig 4:**
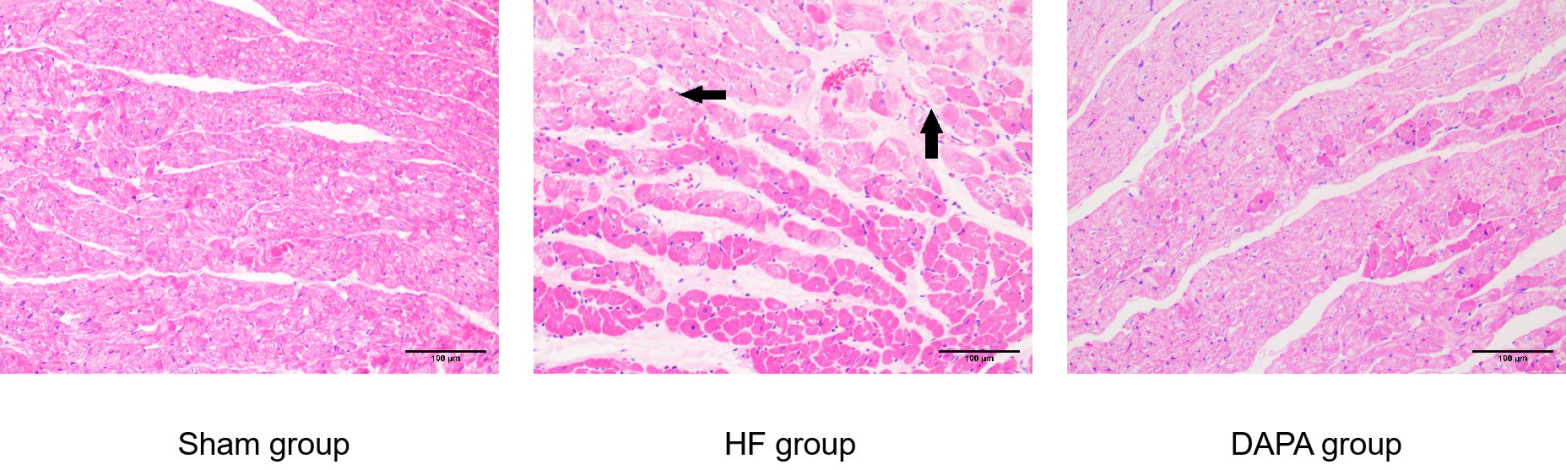
Morphological changes of myocardial tissue in each group of experimental rabbits (HE staining, ×200).

According to the results of HE staining, the myocardial tissue of rabbits in the sham group exhibited normal morphology and structure, with dense and clear myocardial fibers, uniform staining, regular arrangement of myocardial cells, and distinct cross-striations, with no significant infiltration of inflammatory cells in the tissue. Compared to the sham group, the HF group showed alterations in myocardial fiber structure and morphology, with loose and irregularly arranged myocardial cells, cellular swelling, and visible infiltration of inflammatory cells between the myocardial tissues. Compared to the HF group, the DAPA group showed overall improvement in myocardial tissue morphology, with reduced intercellular spaces and decreased inflammatory infiltration, suggesting that dapagliflozin can improve the myocardial cell structure in rabbits with heart failure (Fig 4)

### 3.3 Comparison of myocardial ferrous ion content among each group of rabbits

**Fig 5:**
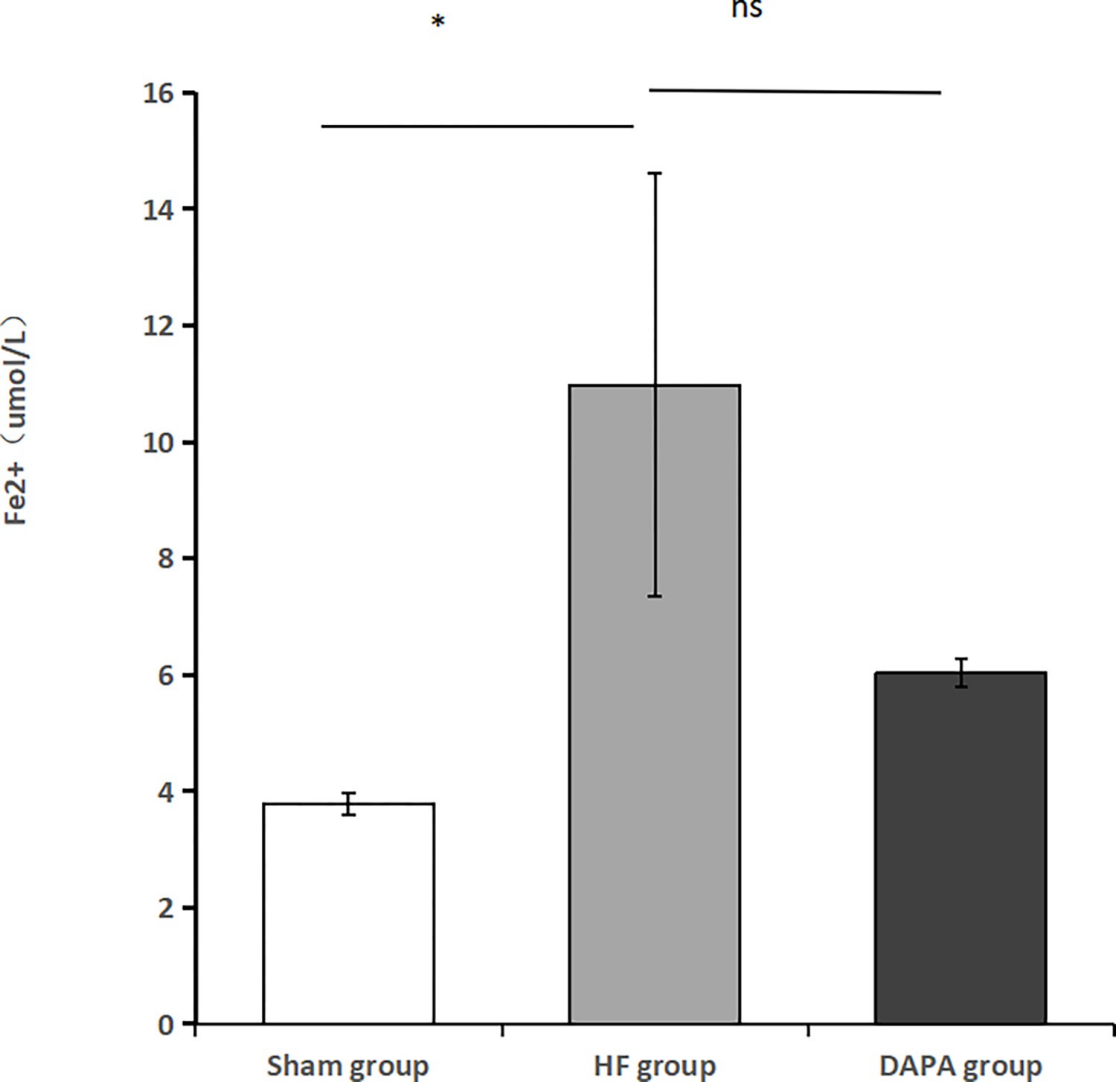
Levels of serum Fe^2+^ content in rabbits in each group as measured by iron kit. Note: *Compared with the Sham group, *P<0.05; ns indicates no statistical significance.

The ferrous ion content in the serum of rabbits in each group did not follow a normal distribution, so a non-parametric test was used. The Kruskal-Wallis test indicated that there was a statistically significant difference in ferrous ion content among the sham group, HF group, and DAPA group (P<0.05). Compared to the sham group, the HF group had a significantly higher serum ferrous ion content (P<0.05). However, compared to the HF group, the DAPA group showed a reduction in serum ferrous ion content, but this difference was not statistically significant (P>0.05) (Table 3, Fig 5).

**Table 3:** Comparison of serum Fe^2+^ levels in rabbits in each group ($\bar{X}$±S).

Group	Sample size	Fe^2+^ (umol/L)
Sham group	3	3.787±0.188
HF group	3	10.977±3.637
DAPA group	3	6.028±0.238

### 3.4 Detection of serum inflammatory factor levels by ELISA

**Fig 6:**
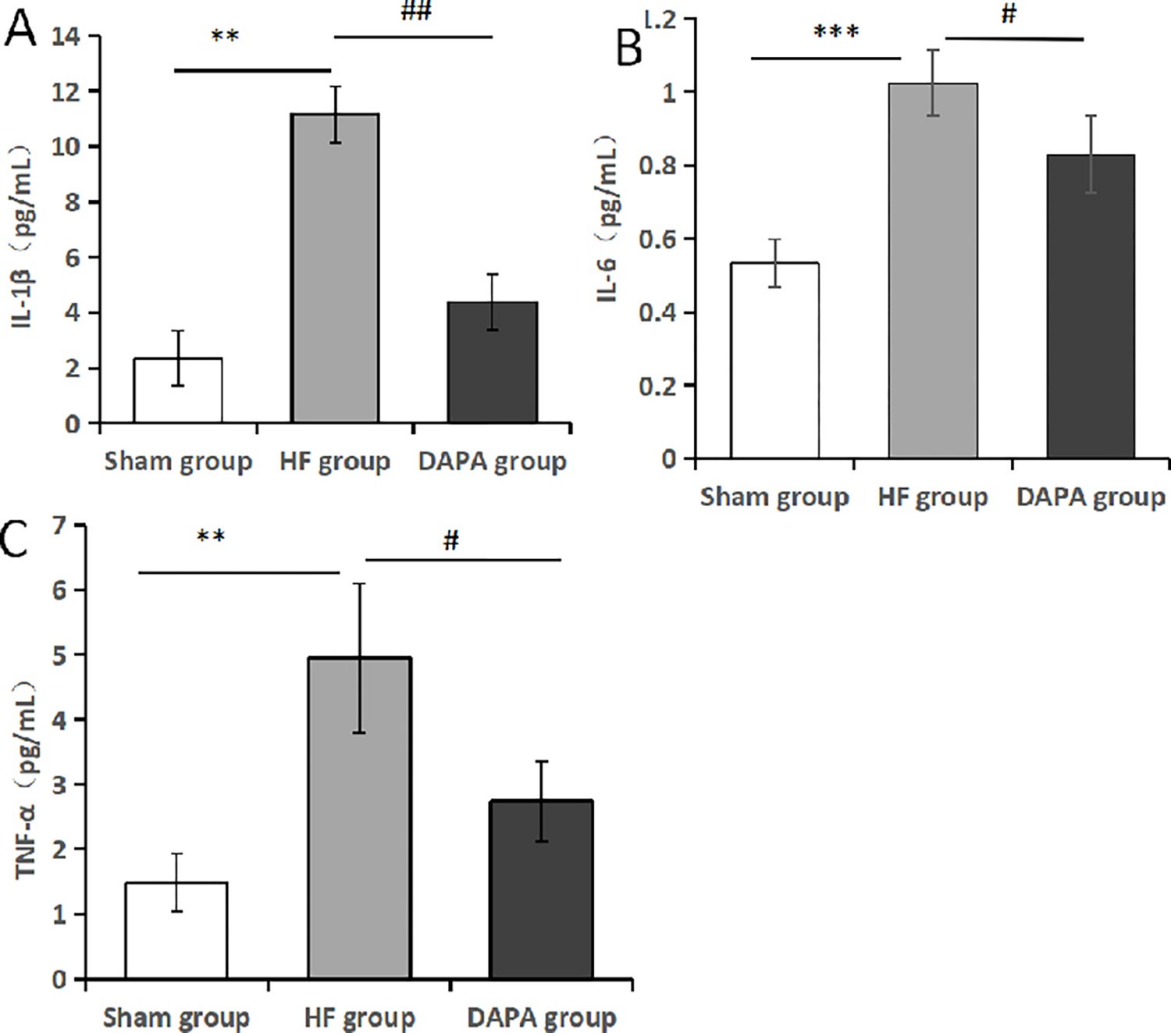
The levels of inflammatory factors were detected by ELISA. Note: (A). IL-1β; (B). IL-6; (C). TNF-α; *Compared with the Sham group, *P<0.05, **P<0.01, ***P<0.001; #compared with the HF group, #P<0.05, ##P<0.01, ###P<0.001.

Compared with sham group, serum levels of IL-1β, IL-6 and TNF-α inflammatory factors in HF group were significantly increased (P<0.05). Compared with HF group, IL-1β, IL-6 and TNF-α inflammatory factors in blood of rabbits with heart failure were decreased in different degrees after administration of dapagliflozin, and there were significant differences (P<0.05) (Table 4, Fig 6).

**Table 4:** Comparison of serum levels of IL-1β, IL-6 and TNF-α in each group ($\bar{X}$±S).

Group	Sample size	IL-1β (pg/mL)	IL-6 (pg/mL)	TNF-α (pg/mL)
Sham group	3	2.34±0.71	0.533±0.065	1.481±0.448
HF group	3	11.15±5.09*	1.025±0.089*	4.948±1.153*
DAPA group	3	4.38±1.38^#^	0.83±0.104^#^	2.741±0.613^#^
F		6.76	24.04	14.532
P		0.029	0.001	0.005

Note: Compared with the Sham group

*p<0.05; compared with the HF group

#p<0.05.

### 3.5 Detection of serum oxidative stress related indexes by colorimetric method (SOD, MDA, GSH-Px)

**Fig 7:**
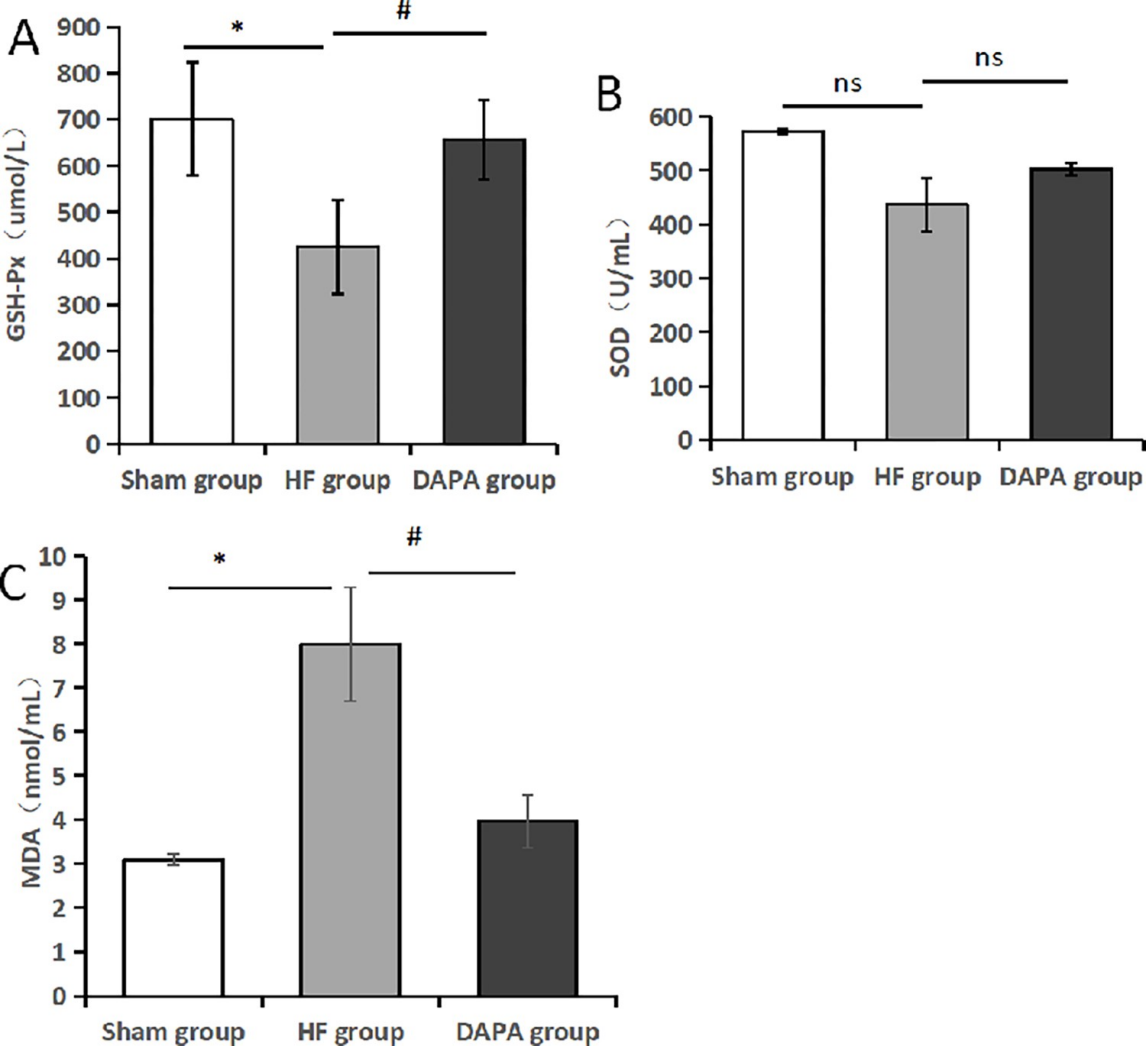
The levels of GSH-Px, SOD and MDA levels in each group. Note: (A). GSH-Px; (B). SOD; (C). MDA; *Compared with the Sham group, *P<0.05; #compared with the HF group, #P<0.05; ns indicates no statistical significance.

Compared with sham group, the serum GSH in HF group was decreased, the difference was statistically significant (P<0.05). Compared with HF group, GSH level in DAPA group was increased, and the difference was statistically significant (P<0.05). Compared with sham group, the serum SOD level of rabbits in HF group was decreased, and the difference was not statistically significant (P>0.05). Compared with HF group, SOD level in DAPA group was increased, but the difference was not statistically significant (P>0.05). Compared with sham group, the serum MDA level in HF group was increased, and the difference was statistically significant (P<0.05). Compared with HF group, MDA level in DAPA group was decreased, the difference was statistically significant (P<0.05) (Table 5, Fig 7).

**Table 5:** Comparison of serum GSH-Px, SOD and MDA levels in each group ($\bar{X}$±S).

Group	Sample size	GSH-Px (umol/L)	SOD (U/mL)	MDA (nmol/mL)
Sham group	3	701.333±122.464	572.986±4.007	3.095±0.113
HF group	3	424±102.137*	436.752±49.238	7.992±1.286*
DAPA group	3	656±86.533^#^	502.198±12.242	3.967±0.602^#^

Note: Compared with the Sham group

*P<0.05; compared with the HF group

#P<0.05.

### 3.6 The protein expression levels of Nrf2, HO-1 and GPX4 were detected by Western blot

**Fig 8:**
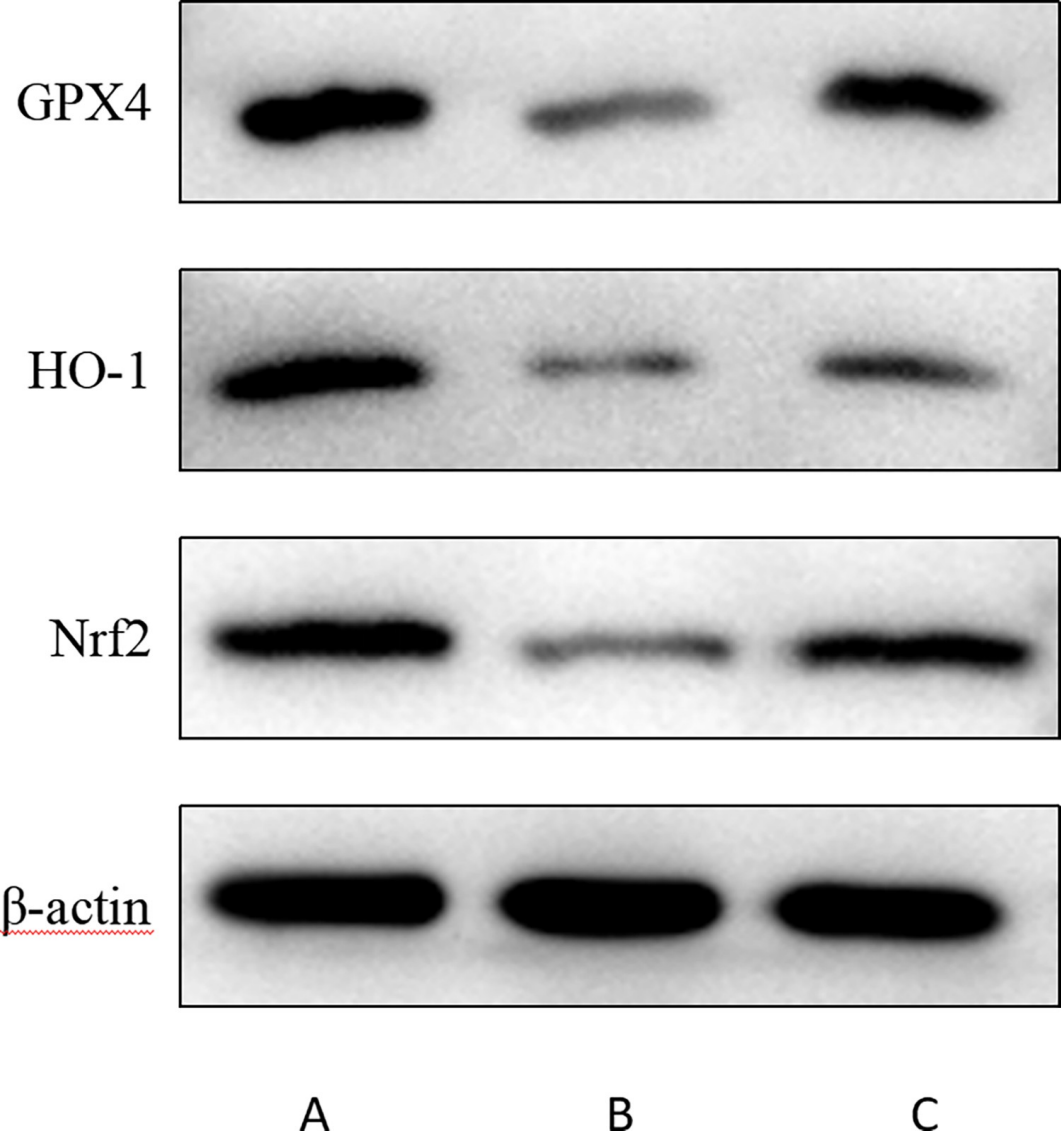
The protein expressions of Nrf2, HO-1 and GPX4 in myocardial tissue of rabbits were detected by WB.

**Fig 9:**
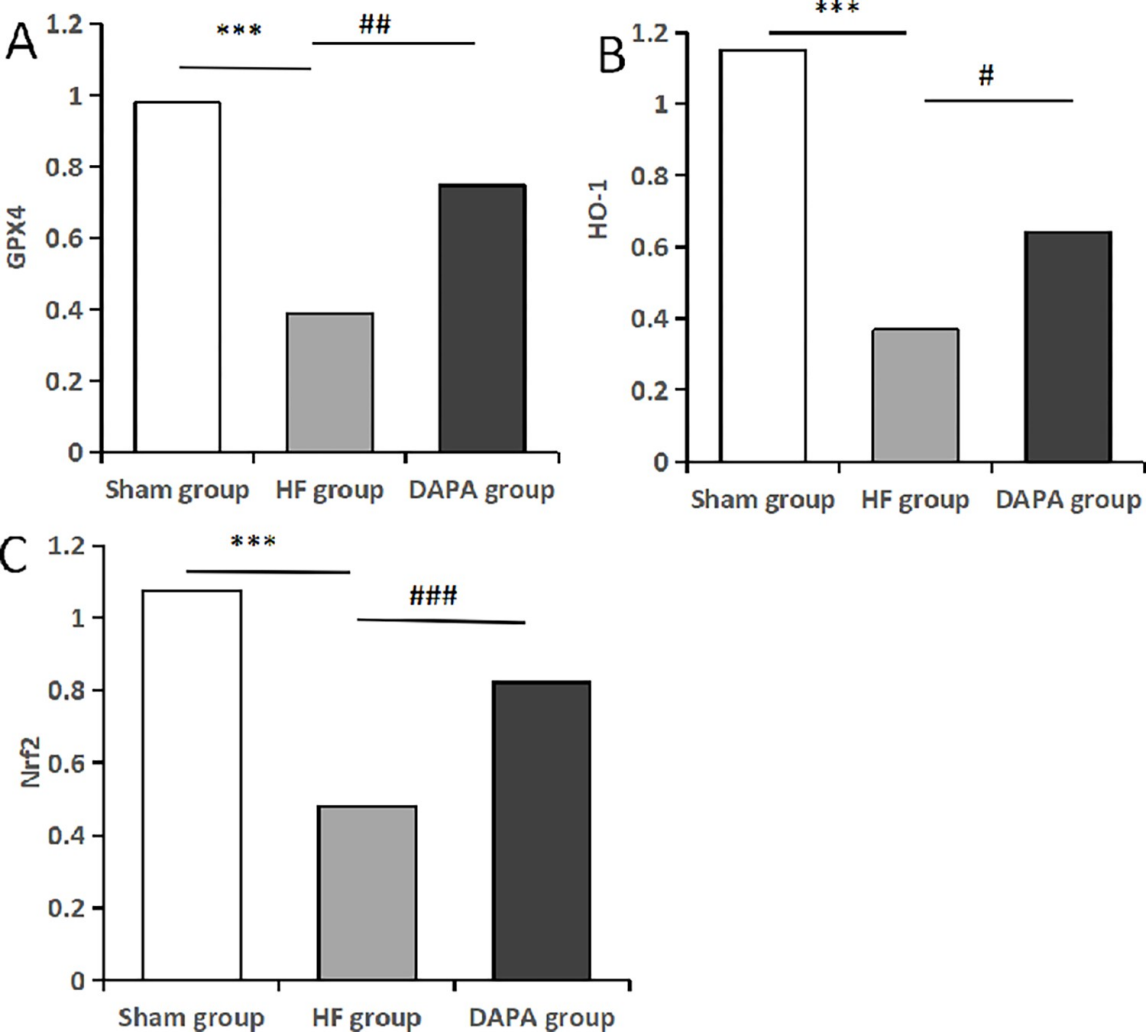
Protein expression levels of Nrf2, HO-1 and GPX4 in each group. Note: (A). GPX4; (B). HO-1; (C). Nrf2; *Compared with the Sham group, *P<0.05, **P<0.01, ***P<0.001; #compared with the HF group, #P<0.05, ##P<0.01, ###P<0.001.

Western blot method was used to study the expression of core target protein in the myocardium of experimental rabbits in each group, and the observation results showed that Nrf2, HO-1 and GPX4 were expressed in different degrees in the heart tissues of the three groups (Fig 8). The results showed that compared with sham group, the expressions of Nrf2, HO-1 and GPX4 in HF group and DAPA group were significantly decreased, and the differences were statistically significant (P<0.05). Compared with HF group, the expressions of Nrf2, HO-1 and GPX4 in myocardium of DAPA group were significantly increased, and the differences were statistically significant (P<0.05) (Table 6, Fig 9).

**Table 6:** Comparison of serum Nrf2, HO-1, GPX4 protein expression levels in each group ($\bar{X}$±S).

Group	Sample size	GPX4	HO-1	Nrf2
Sham group	3	0.979±0.075	1.150±0.04	1.075±0.048
HF group	3	0.39±0.732*	0.368±0.133*	0.482±0.082*
DAPA group	3	0.746±0.042^#^	0.643±0.188^#^	0.822±0.044^#^
F		62.105	25.992	72.353
P		0.000	0.001	0.000

Note: Compared with the Sham group

*P<0.05; compared with the HF group

#P<0.05.

## 4 Discussion

Heart failure is the end-stage clinical manifestation of cardiovascular disease, and its incidence increases significantly with the increase of age and the acceleration of population aging. Current research shows that the incidence of HF in adults is about 2%. As people age, the incidence of HF rises from 1% at age 55 to 10% at age 70 [11, 12]. Inflammation and oxidative stress have been identified as key mechanisms leading to cardiovascular dysfunction [13]. In addition to various cell death modes, such as apoptosis, autophagy and necrosis, which have been confirmed to participate in the process of heart failure, ferroptosis is also involved. Ferroptosis of cardiomyocytes is involved in the pathogenesis of heart failure in some cases. Previous studies have found that the lipid hydroperoxide and unstable iron pool in cardiomyocytes are increased, and the expression of GPX4 and ferritin is down-regulated, indicating that ferroptosis occurs in cardiomyocytes [14]. At present, the main objective of clinical treatment of CHF is to improve cardiac function and reduce the risk of acute attack and death [15]. At present, although there are many methods to treat HF, they have not significantly reduced the mortality and hospitalization rate caused by HF. Therefore, new therapeutic targets should be actively explored, so as to improve the prognosis of patients and reduce the hospitalization rate and morbidity.

Dapagliflozin is one of the representative drugs of SGLT2-i, which is an oral hypoglycemic drug and has been widely used in the clinical treatment of type II diabetes patients. Dapagliflozin is the first SGLT2-i to be marketed in China [16]. Selective SGLT2-i can inhibit the reabsorption of glucose and sodium by the proximal renal tubules, and promote the secretion of glucose into urine, thereby regulating the blood glucose concentration in diabetic patients and playing a role in lowering blood glucose [17, 18]. The study found that SGLT2-i have an additional cardiovascular protective effect and reduce the incidence of cardiovascular death, hospitalization, and emergency visits due to heart failure in patients with heart failure [19, 20]. In this study, a rabbit model of chronic heart failure was successfully established using coarctation of the aorta. The effects of dapagliflozin on ventricular structure and function, as well as related hematologic indexes of HF in CHF rabbits were observed by force-feeding, and the specific mechanism of improving heart failure by this class of drugs was further explored.

Previous studies have shown that a large number of inflammatory factors such as IL-6 and TNF-α are expressed in the serum of patients with heart failure [21]. The increase of IL-6 content can promote the apoptosis of cardiomyocytes, and TNF-α is an important index reflecting the degree of heart failure [22]. As an inflammatory cascade reaction, inflammatory factor IL-1β plays a major regulatory role in the occurrence and development of various cardiovascular diseases. In this study, IL-1β, IL-6, TNF-α and other inflammatory factors were significantly increased in the HF group compared with the sham group (P<0.05), indicating the existence of inflammation in rabbit myocardial tissue during HF. Compared with HF group, the levels of IL-1β, IL-6 and TNF-α inflammatory factors in DAPA group were decreased (P<0.05). These experimental results suggest that Dapagliflozin can play an inflammatory inhibitory role in anti-heart failure therapy.

Oxidative stress, which is closely related to the development of HF and its comorbidities, is a metabolic imbalance in which the production of oxygen free radicals in the body increases over its clearance. At the same time, oxidative stress is also involved in the mechanism of ferroptosis. MDA is one of the main products of lipid peroxidation, and its content can directly reflect the degree of lipid peroxidation, and indirectly reflect the degree of myocardial damage caused by free radical attack on cardiomyocytes; SOD and GSH-Px play an important role in the body’s antioxidant system, which can remove ROS, oxygen free radicals, hydroxyl free radicals, etc., and maintain the structure and function of the cell membrane. The level of SOD and GSH-PX can indirectly reflect the body’s ability to remove oxygen free radicals and resist lipid peroxidation. Our results showed that compared with the HF group, the serum MDA levels of rabbits in the DAPA group were decreased, and the levels of SOD and GSH were significantly increased (P<0.05), suggesting that dapagliflozin may inhibit ferroptosis through mediating oxidative stress response to achieve the goal of treating CHF.

The regulatory mechanism of ferroptosis is complex, mainly involving iron metabolism, amino acid metabolism and lipid metabolism. It is due to the dysfunction of phospholipid oxidation metabolism in cell membranes. When the accumulation of lipid peroxides reaches the reducing limit of glutathione peroxidase, the Fenton reaction mediated by iron ions will catalyze the production of lipid free radicals, which will accumulate in large quantities in cells and cause cell death [23]. In the process of ferroptosis, excess iron accumulates in the cell and produces ROS, oxidizes polyunsaturated fatty acids, causes the destruction of cell membrane structure, and ultimately leads to cell death. It is characterized by the accumulation of iron-dependent lipid peroxides, and morphology is mainly manifested by mitochondrial volume reduction, mitochondrial ridge shrinkage, membrane density increase, and outer membrane rupture [24]. Nrf2/HO-1/GPX4, as one of the important pathways regulating ferroptosis, can regulate the metabolism of iron and lipid in cells and inhibit lipid peroxidation, thus protecting cells from ferroptosis [25]. Nrf2 is an important transcription factor that regulates cellular oxidative stress and a key negative regulator of ferroptosis. Previous studies have found that the expression of GPX4 and ferritin is down-regulated, indicating ferroptosis in cardiomyocytes [14]. Nrf2 is activated by nuclear translocation under oxidative stimulation, which activates the expression of downstream antioxidant genes such as HO-1 and GPX4, regulates iron metabolism and lipid metabolism, reduces inflammation and oxidative stress, and alleviates ferroptosis. Our results showed that compared with sham group, the protein expression levels of Nrf2, HO-1 and GPX4 in myocardial tissue of rabbits in HF group were significantly decreased. Compared with HF group, the protein expression levels of Nrf2, HO-1 and GPX4 in myocardium of rabbits in DAPA group were significantly increased. Based on these findings, it can be concluded that dapagliflozin improves HF by inhibiting ferroptosis, which may be achieved by inhibiting oxidative stress and reducing iron content by regulating Nrf2/HO-1/ GPX4 signaling pathway.

To sum up, this study was based on the rabbit model of heart failure established by ascending aorta ligation to observe the effect of dapagliflozin on HF. The results showed that dapagliflozin can improve chronic heart failure, and dapagliflozin can improve CHF by inhibiting ferroptosis in rabbits. The mechanism may be to inhibit ferroptosis by activating Nrf2/HO-1/GPX4 signaling pathway to inhibit oxidative stress. In this study, the ameliorative effect of dapagliflozin on HF was discussed from the perspective of ferroptosis. However, due to the complex pathogenesis of HF and the complex regulatory mechanism of ferroptosis, whether dapagliflozin can play a myocardial protective role through other pathways or targets still needs to be further explored.

## Supporting information

S1 Raw images (PDF)

S1 Raw data (XLSX)
