# Supplementary figures and images for: Dapagliflozin inhibits ferroptosis to improve chronic heart failure by regulating Nrf2/HO-1/GPX4 signaling pathway
Source: PLoS One. 2025 Jan 28;20(1):e0317295. doi: 10.1371/journal.pone.0317295 (PMC11774390; doi:10.1371/journal.pone.0317295)

|       | 14V                                                                                 | 8                                                                                   | 3                                                                                   |  | 17V                                                                                  | 10                                                                                    | 13                                                                                    |  | 15V                                                                                   | 14                                                                                    | 24                                                                                    |  |
|-------|-------------------------------------------------------------------------------------|-------------------------------------------------------------------------------------|-------------------------------------------------------------------------------------|--|--------------------------------------------------------------------------------------|---------------------------------------------------------------------------------------|---------------------------------------------------------------------------------------|--|---------------------------------------------------------------------------------------|---------------------------------------------------------------------------------------|---------------------------------------------------------------------------------------|--|
| GPX4  | 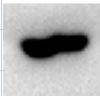   | 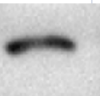   | 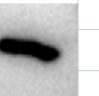   |  | 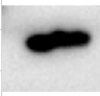   | 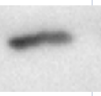   | 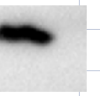   |  | 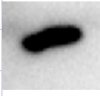   | 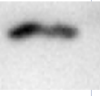   | 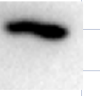   |  |
|       | 26174                                                                               | 12809                                                                               | 21565                                                                               |  | 26053                                                                                | 8593                                                                                  | 19361                                                                                 |  | 27838                                                                                 | 11419                                                                                 | 19001                                                                                 |  |
| ACTIN | 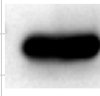   | 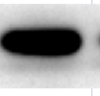   | 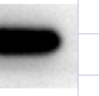   |  | 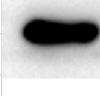   | 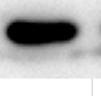   | 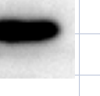   |  | 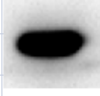   | 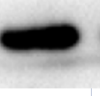   | 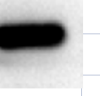   |  |
|       | 27457                                                                               | 29273                                                                               | 27608                                                                               |  | 28330                                                                                | 28099                                                                                 | 27647                                                                                 |  | 26178                                                                                 | 26753                                                                                 | 25070                                                                                 |  |
| ho-1  | 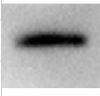   | 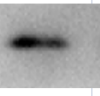   | 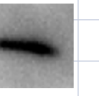   |  | 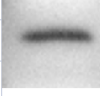   | 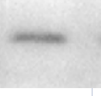   | 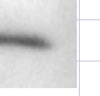   |  | 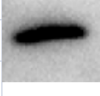   | 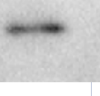   | 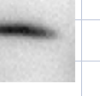   |  |
|       | 28482                                                                               | 12472                                                                               | 21507                                                                               |  | 27397                                                                                | 5624                                                                                  | 12310                                                                                 |  | 30929                                                                                 | 8846                                                                                  | 13106                                                                                 |  |
| ACTIN | 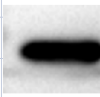   | 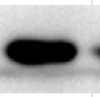   | 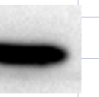   |  | 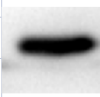   | 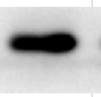   | 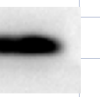   |  | 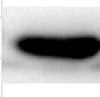   | 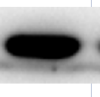   | 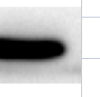   |  |
|       | 25496                                                                               | 24656                                                                               | 25092                                                                               |  | 24051                                                                                | 23341                                                                                 | 24388                                                                                 |  | 25877                                                                                 | 24790                                                                                 | 23087                                                                                 |  |
| nrf2  | 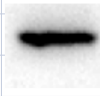 | 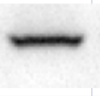 | 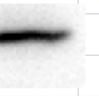 |  | 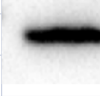 | 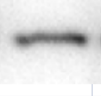 | 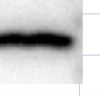 |  | 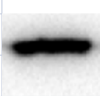 | 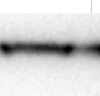 | 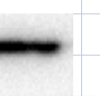 |  |
|       | 27953                                                                               | 13782                                                                               | 20695                                                                               |  | 30948                                                                                | 11130                                                                                 | 23305                                                                                 |  | 30022                                                                                 | 15857                                                                                 | 25581                                                                                 |  |
| ACTIN | 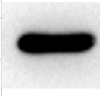 | 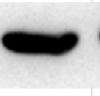 | 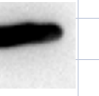 |  | 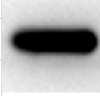 | 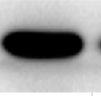 | 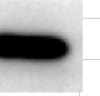 |  | 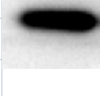 | 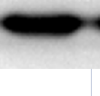 | 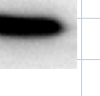 |  |
|       | 25883                                                                               | 25362                                                                               | 26780                                                                               |  | 27598                                                                                | 28674                                                                                 | 27192                                                                                 |  | 29292                                                                                 | 30897                                                                                 | 30592                                                                                 |  |

Supplement: S1 Raw images — (PDF) [file pone.0317295.s001.pdf]
